# Non-invasive stroke volume assessment in patients with pulmonary arterial hypertension: left-sided data mandatory

**DOI:** 10.1186/1532-429X-10-51

**Published:** 2008-11-05

**Authors:** Gert-Jan Mauritz, J Tim Marcus, Anco Boonstra, Pieter E Postmus, Nico Westerhof, Anton Vonk-Noordegraaf

**Affiliations:** 1 Department of Pulmonary Diseases, Institute for Cardiovascular Research ICaR-VU, VU University Medical Center, Amsterdam, The Netherlands; 2 Department of Physics and Medical Technology, Institute for Cardiovascular Research ICaR-VU, VU University Medical Center, Amsterdam, The Netherlands; 3 Department of Physiology, Institute for Cardiovascular Research ICaR-VU, VU University Medical Center, Amsterdam, The Netherlands

## Abstract

**Background:**

Cardiovascular Magnetic Resonance (CMR) is an emerging modality in the diagnosis and follow-up of patients with Pulmonary Arterial Hypertension (PAH). Derivation of stroke volume (SV) from the pulmonary flow curves is considered as a standard in this respect. Our aim was to investigate the accuracy of pulmonary artery (PA) flow for measuring SV.

**Methods:**

Thirty-four PAH patients underwent both CMR and right-sided heart catheterisation. CMR-derived SV was measured by PA flow, left (LV) and right ventricular (RV) volumes, and, in a subset of nine patients also by aortic flow. These SV values were compared to the SV obtained by invasive Fick method.

**Results:**

For SV by PA flow versus Fick, r = 0.71, mean difference was -4.2 ml with limits of agreement 26.8 and -18.3 ml. For SV by LV volumes versus Fick, r = 0.95, mean difference was -0.8 ml with limits of agreement of 8.7 and -10.4 ml. For SV by RV volumes versus Fick, r = 0.73, mean difference -0.75 ml with limits of agreement 21.8 and -23.3 ml. In the subset of nine patients, SV by aorta flow versus Fick yielded r = 0.95, while in this subset SV by pulmonary flow versus Fick yielded r = 0.76. For all regression analyses, p < 0.0001.

**Conclusion:**

In conclusion, SV from PA flow has limited accuracy in PAH patients. LV volumes and aorta flow are to be preferred for the measurement of SV.

## Introduction

In pulmonary arterial hypertension (PAH), cardiovascular magnetic resonance (CMR) has been proposed as a standard for the assessment of right ventricular function and characteristics of the pulmonary vascular bed [1,2]. Accurate assessment of stroke volume (SV) by CMR is critical in this respect, since earlier studies revealed that SV is closely related to prognosis and that a change in SV reflects treatment effects [3,4]. Since most of the CMR protocols used in PAH [5,6] measured pulmonary artery flow, SV can be assessed by measuring flow in the main pulmonary artery (PA).

Previous studies have shown that this method is accurate to measure SV from PA flow in healthy subjects [7-9]. Whether this also holds true in PAH is questionable, since the velocity profile in PAH is non-laminar, in contrast to the profile in healthy subjects [10-13].

For this test of accuracy, a clinical standard is required. This standard is provided by the measurement of SV by the direct Fick principle during right heart catheterisation (RHC) [14]. However, this is an invasive procedure and thus not well suited for either screening or frequently repeated follow-up measurements.

Therefore the aim of the present study is to assess the accuracy of the PA flow by CMR for measuring SV in PAH patients, by comparing SV from this PA flow with the SV assessed by the Fick method. In addition, other CMR-derived SV measures from aorta flow and cine imaging in the same patients will also be compared versus the Fick-derived SV.

## Materials and methods

### Patients

This study was approved by the institutional Review Board on Research Involving Human Subjects of the VU University medical centre, and all participants gave written informed consent. Between January 2004 and April 2007, a total of 34 patients who were given a final diagnosis of PAH after a complete diagnostic workup including RHC, underwent CMR. RHC and CMR were performed within 12 hours. The study group consisted of 23 female (68%) and 11 male (32%) patients with a mean age of 45 years ± 17 (standard deviation), and an age range of 20–84 years.

### CMR imaging protocol

#### CMR flow measurements

SV was measured using both phase contrast flow and volumetric methods as described below.

CMR was perforned with a Siemens 1.5 T 'Sonata' whole body scanner (Siemens Medical Solutions, Erlangen. Germany), equipped with a phased-array body coil.

Phase-contrast CMR was acquired during continuous breathing with a gradient echo MR sequence, with velocity encoding perpendicular to the imaging plane and a velocity sensitivity of 120 cm/sec. The flow sequence was run with the following parameters: orientation = orthogonal to the main PA, slice thickness = 6 mm, field of view = 240 × 320 mm^2^, matrix size = 140 × 256, echo time = 4.8 ms, repetition time = 11 ms, temporal resolution = 22 ms, flip angle = 25°.

To explore whether there is inaccurate determination of flow-derived SV due to inherent technical limitations of phase-contrast CMR, aortic flow was also measured in a subset of nine patients, approximately 2 – 4 cm above the aortic valve and distal to the coronary arterial ostia, and the aortic flow-derived SV was also compared to the Fick-derived SV.

After the flow images were acquired, a 7 litre bottle containing H2O, with per litre 1.25 g NiSO4.6H2O + 2.6 g NaCL ("phantom") was then imaged with identical imaging parameters, to serve as correction for the background phase error in the mean PA and Aorta[15].

#### RV and LV volumetric measurements

The short-axis slices needed to encompass the entire left and right ventricle volumes to measure right ventricular (RV) and left ventricular (LV) volumes-derived SV, were obtained by steady state free precession imaging: 11 phase encoding lines per heart beat (which means 11 lines per segment), flip angle = 60°, slice thickness = 6 mm, slice gap = 4 mm, temporal resolution 36.3 ms and retrospectively ECG gated. Acquisition was in breathhold, acquisition time was 14 heartbeats.

### Imaging analysis

#### CMR flow measurements

CMR post-processing was performed using the 'FLOW' software package (Dept. of Radiology, Leiden University Medical Center, Leiden, The Netherlands). The heart rate during cardiac CMR was recorded from the images. No aliasing due to high peak systolic velocities was encountered. The contours of the mean pulmonary artery MPA were automatically traced, with manual correction when necessary, simultaneously on magnitude and velocity-map images of all reconstructed phases. The software then calculated the velocity in each of the pixels included within the contours. The flow in each pixel (velocity times pixel area) is calculated and the pixel flow within the contour is summed. This is done for every temporal phase, resulting in blood flow as a function of time through the main PA.

**Figure 1:**
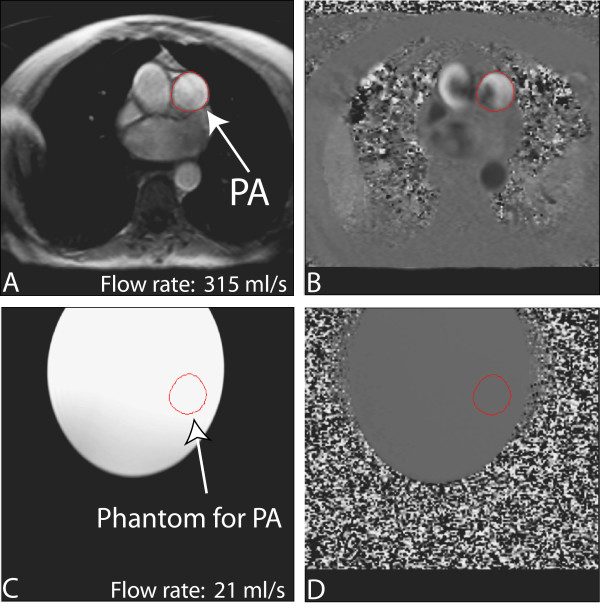
**Double-oblique gradient-echo phase-contrast CMR (repetition time ms/echo time msec 11/4.8; flip angle 25 deg; section thickness, 6 mm; matrix, 140 × 256), perpendicular to pulmonary trunk.** Region of interest (ROI) placement is shown for measuring pulmonary artery (PA) flow. In (a) magnitude and (b) velocity image is shown. The images correspond to the time frame when maximum flow is measured in the PA. ROI placement for the corresponding phantom magnitude (c) and velocity (d) image is shown to correct velocity offset error in PA.

**Figure 2:**
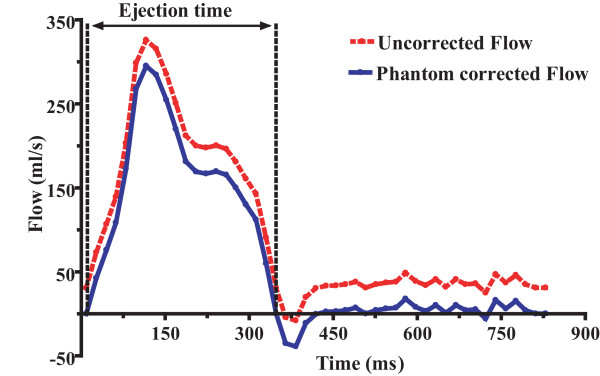
**Flow curve of the main pulmonary artery (MPA) during a cardiac cycle in a PAH patient.** Uncorrected stroke volume (SV) is obtained by integration of the flow curve during ejection time and is 58 ml, ejection time is 0.323s and phantom offset value is 21.13 ml/s. Phantom corrected SV is (58-(0.323*21.13)) = 51 ml.

Following image acquisition, pulmonary blood flow was corrected using the offset values from the phantom (figure 1). After background correction with the phantom (figure 2), we calculated PA forward flow volume as the area under the curve until the zero-crossing of the downward limb. We checked whether there was more than 5% reverse flow that would indicate any pulmonary regurgitation (PR). However we did not observe any PR, thus we did not exclude any PR patient. The same was done for the Aorta flow.

#### RV and LV volumetric measurements

**Figure 3:**
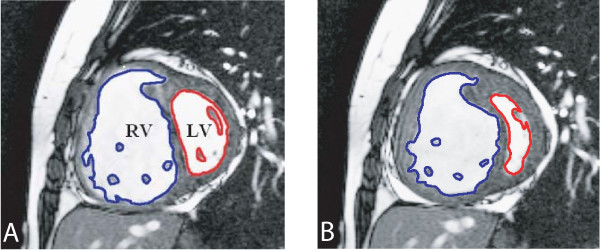
**Double-oblique steady-state free precession cine MR images (repetition time ms/echo time ms 3.3/1.65; flip angle 60 deg; section thickness, 6 mm; matrix, 256 × 150).** End-diastolic (a) and end-systolic (b) cardiac short axis slice in PAH patient. The endocardial boundaries of the right ventricle (RV) and left ventricle (LV) are traced for calculation of end-diastolic volume (EDV) and end-systolic volume (ESV). EDV and ESV were calculated by summation of the product (area × slice distance) for all slices. SV is then given by SV = EDV-ESV for RV and LV.

We measured RV and LV volumes as follows [16]. The endocardial contours of the ventricles were manually traced on short-axis slices in end-diastolic (first cine phase of the R-wave triggered acquisition) and end-systolic (image phase with smallest cavity area) phases using commercial software (MASS software package, version 5.0,) (figure 3). In the present study, papillary muscles were excluded from manual tracings of the endocardial contours of the right end left ventricle. The ventricular areas were then measured and ventricular volumes calculated by adding the ventricular areas and multiplying by the slice distance. End-Diastolic Volumes (EDV) and End-Systolic Volumes (ESV) were used to calculate LV-and RV volumes-derived SV. One investigator analyzed both phase contrast Flow and volumetric images unaware of stroke volume values measured by direct Fick principle.

### Right Heart Catheterization (RHC)

Diagnostic right heart catheterization was performed with a balloon tipped, flow directed 7F Swan-Ganz catheter (131HF7; Baxter Healthcare Corp; Irvine, CA). The direct Fick Principle (DF) was used. The patient was in stable condition, lying supine and breathing room air. Right atrial, right ventricular, pulmonary artery and pulmonary capillary wedge pressures were measured. Heart rate was monitored continuously. Average steady state oxygen consumption was obtained at the same time during RHC using a timed collection of expired air to measure oxygen consumption by an on-line analyzer (Vmax 229, Sensormedics, Yorba Linda, USA) connected to a mouth piece. To ensure accuracy, the system was calibrated before each study and the values were time-averaged over at least 5 minutes. Simultaneous arterial and mixed venous blood samples were then drawn for measurement of arterial oxygen saturation (SaO_2_) and haemoglobin (Hb) concentration. Arterial blood samples were obtained through either a radial or femoral arterial puncture and the mixed venous blood samples were obtained from the distal port of the pulmonary artery catheter. The cardiac output (CO) was then calculated by dividing the average oxygen consumption (VO_2_) value with the difference between the concentration of arterial oxygen (CaO_2_) and concentration of mixed venous oxygen content (CvO_2_). Eventually, SV (ml) was determined by dividing CO (L/min) by HR (beats/min).

### Intra-observer and inter-observer variability

Interobserver variability of the different CMR methods was assessed by a second investigator analyzing all of data sets. Intraobserver variability was analyzed by assessing SVs obtained by the different CMR methods twice by one observer. The two assessments were separated by a one month period, and the observer was blinded for his previous results.

### Data analysis

The results are expressed as mean ± SD. For each measurement of SV, the values for SV obtained invasively direct Fick and by the CMR-based methods were compared by regression analysis. Bland-Altman analysis was used to compare the degree of agreement between the three CMR methods and the Fick method for SV measurement [17]. Bias was defined as the mean value of the differences between CMR methods and the Fick principle. Precision was defined as 1 standard deviation (SD) of the differences and limits of agreement as the bias ± 2 SDs reported as millilitres. The percentage error was calculated as the ratio of 2 times the SD to mean SV. Tendency toward over- or underestimation of SV by the different CMR-based methods was assessed with the two-tailed Student's *t*-test for paired data.

The mean difference (bias) and the coefficient of variability (CoV = SD of repeated measures as % of their mean) were used to assess intra- and interobserver variability.

P values less than .05 were considered to indicate significant differences. All statistical analyses were performed using GraphPad PRISM (version 4.0, GraphPad software, San Diego, CA).

## Results

The demographic data, causes of PAH and resting clinical hemodynamic measurements obtained at RHC of the 34 PAH patients are shown in Table 1. Twenty-nine patients (out of 34) had measurable Tricuspid Regurgitation by echo, and 4 patients had right-to-left shunting based on the passage of contrast bubbles through a patent foramen ovale, observed by echo-cardiography.

**Table 1:** Baseline characteristics of the patients*

Characteristic	Value
*No of PAH patients*	n = 34
Idiopathic PAH	n = 14
Familial PAH	n = 6
PAH and Collagen vascular disease	n = 5
PAH and HIV infection	n = 6
PAH and Porto pulmonary syndrome	n = 3
Age, yr	54 ± 17
Male/female	11/23
*Functional status:*	
NYHA II	n = 18
NYHA III	n = 12
NYHA IV	n = 4
6 min walk test, m	441 ± 109
*Hemodynamic variables:*	
Pra, mmHg	7 ± 4.5
sPap, mmHg	74 ± 21
dPap, mmHg	29 ± 11
mPap, mmHg	45 ± 10
Pcwp, mmHg	6.9 ± 3.3
SaO_2_, %	95 ± 2
SvO_2_, %	63 ± 8
Q-Fick, l/min	4.7 ± 1.3
CI, l·min^-1^·m^-2^	2.5 ± 0.6
HR, min^-1^	81.5 ± 12
SV-Fick, ml/beat	58.8 ± 16
PVR, dynes·s^-1^·cm^-5^	777 ± 402

PAH = idiopatische pulmonary arterial hypertension; NYHA = modified New York Association; Pra = right atrial pressure; sPap = systolic pulmonary artery pressure; dPap = diastolic pulmonary artery pressure; mPap = mean pulmonary artery pressure; Pcwp = pulmonary capillary wedge pressure; Sao2= arterial oxygen saturation SvO_2 _= mixed venous oxygen saturation; Q-Fick = cardiac output(as estimated by the Fick method; CI = cardiac index; HR = heart rate; SV-Fick = stroke volume PVR = pulmonary vascular resistance; *Values are expressed as mean ± SD

**Figure 4:**
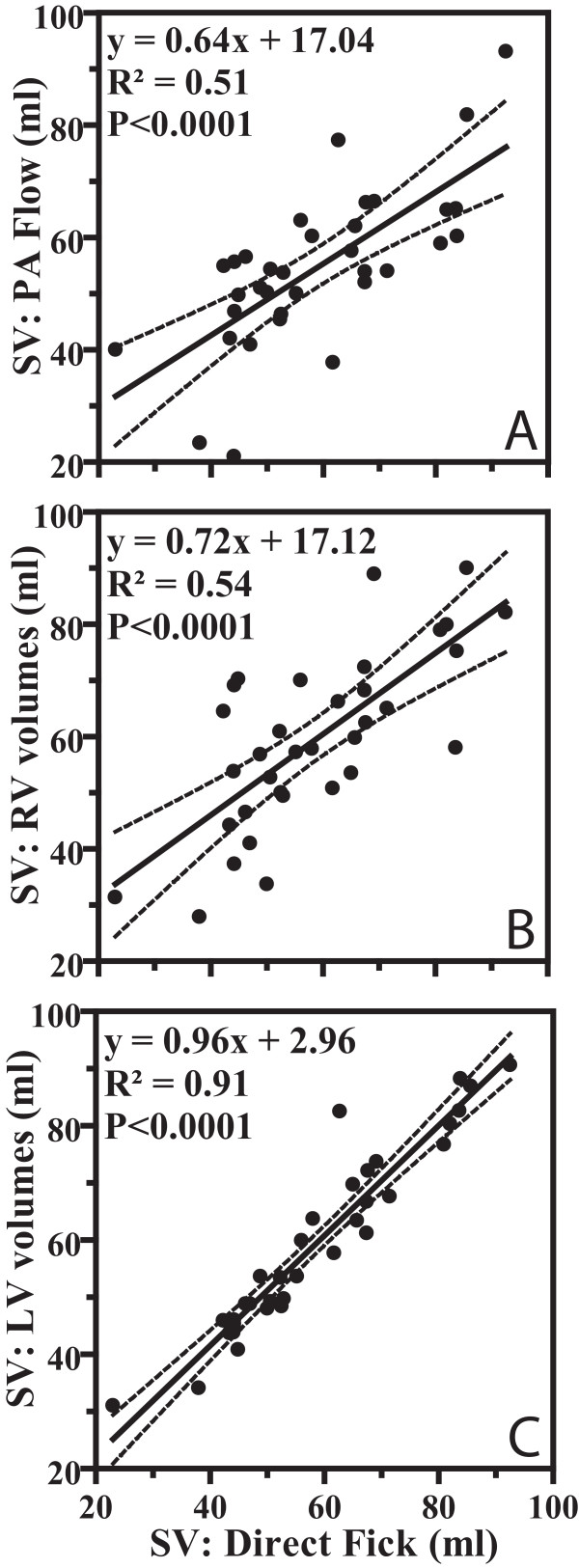
**Linear regression analysis of the correlation between direct Fick and (a) PA flow with phase-contrast MRI, (b) RV volumetric assessment and (c) LV volumetric assessment in 34 PAH patients for stroke volume (SV) (ml).** Dashed lines = 95% CIs.

Figure 4 shows the results of the three different CMR methods to measure stroke volume in comparison with SV measurements assessed by the direct Fick principle. From this figure it is clear that the PA flow-derived SV values and the RV volumes-derived SV values show only a limited correlation with the Fick standard. LV volumes-derived SV showed the best correlation with Fick.

**Figure 5:**
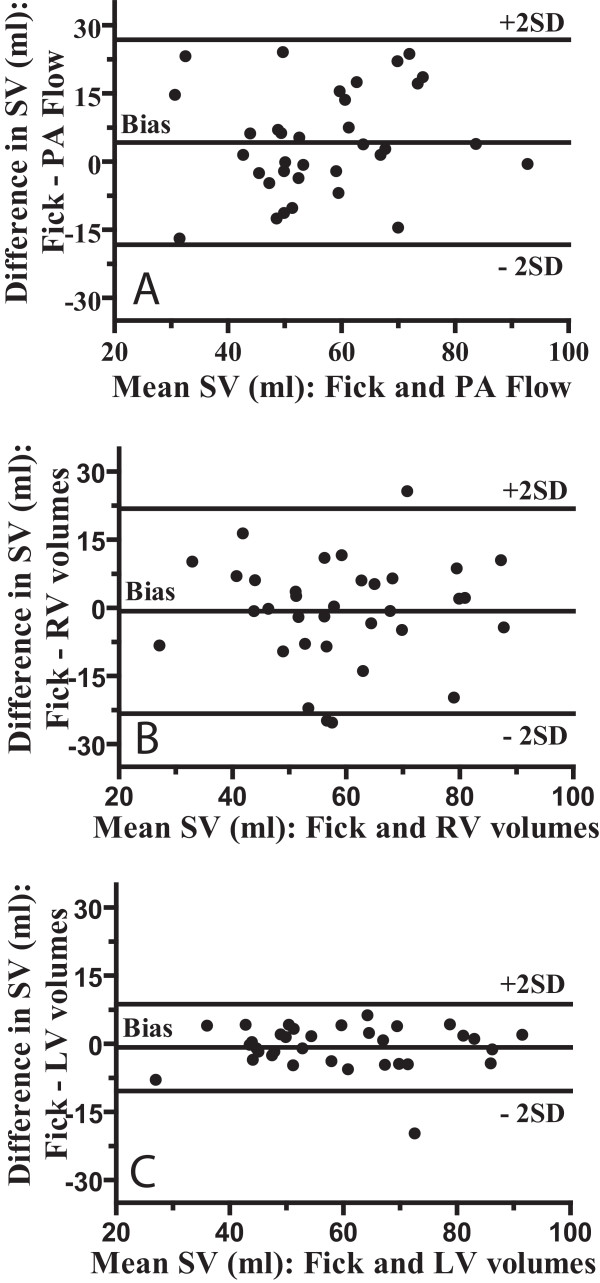
**Bland-Altman plots shows degree of agreement between direct Fick and (a) PA flow, (b) RV volumes and (c) LV volumes in 34 PAH patients.** Central line demonstrates bias; outer lines demonstrate upper and lower limits of agreement (± 2 standard deviations *[SD]*)

Figure 5 shows the Bland-Altman plots of the difference between the CMR based methods and the direct Fick against the mean of both values. Mean (± SD), Mean Difference (bias), SD of the difference (precision), limits of agreement, and percentage error according to different CMR based SV measurements are presented in Table 2. As shown in this Table 2, the limits of agreement of the PA flow-derived SV with Fick present almost the same range of dispersion as was found when RV volumes-derived SV was compared with Fick. The limits of agreement between Fick and LV volumes-derived SV are narrow.

**Table 2:** Results of Bland-Altman analyses of CMR based measurements for stroke volume

	**Mean ± SD**	**Bias**	**Precision**	**95% Limits of Agreement**	**Error (%)**
**PA Flow**	55.2 ± 13.1	-4.2	11.48	-18.3 to 26.8	42%
Aorta Flow*	51.40 ± 13.3	-2.3.	3.85	-9.8 to 5.2	14%
**RV volumes**	59.5 ± 15.7	-.75	11.49	-23.3 to 21.8	39%
**LV volumes**	59.6 ± 16.2	-0.8	4.87	-10.4 to 8.8	16%

NOTE. Values are ml, PA = Pulmonary artery, RV = right ventricle, LV = Left ventricle. *Aorta flow in nine patients.

Bland-Altman analysis demonstrated a small degree of underestimation of SV by PA flow with mean difference of 4.2 ml, the underestimation was statistically significant (P = 0.039).

If those patients with echo-derived right-to-left shunting were excluded, than the correlation coefficient between PA Flow-derived SV and Fick-derived SV increased to r^2 ^= 0.56 and the bias decreased to a non-significant level of 1.9 ± 3.7 ml.

The slight overestimation of SV by RV and LV volumes, 0.75 ml and 0.8 ml respectively, was not significant.

**Figure 6:**
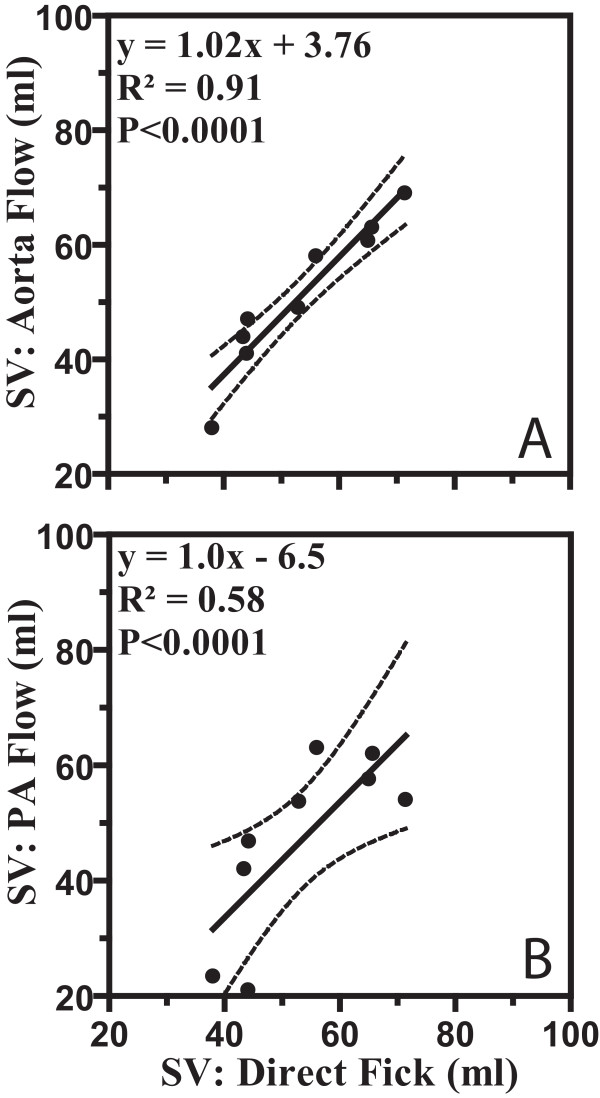
**In a subset of nine patients both aorta and pulmonary flow was measured showing excellent correlation between the direct Fick and the (a) aorta flow in contrast to the direct Fick and the (b) pulmonary flow for stroke volume (SV).** Dashed lines = 95% CIs.

The SV assessed from the aortic flow (figure 6a) in the subset of 9 patients showed an tight relation with the Fick-derived SV. The comparison with Fick was also made for the PA flow-derived SV, in the same subset of patients (figure 6b).

Table 3 displays the intra-observer and inter-observer variability data. Intra-observer and interobserver bias for PA flow (0.8 – 0.9 ml), as well as for Aorta flow (0.7–1.3 ml), RV volumes (2.1–2.6 ml) and LV volumes (1.8–2.57 ml), were negligible. Intra-observer and inter-observer variability was sufficiently low for all different CMR methods for SV assessment, as shown by CoV values (2.7–13.3%).

**Table 3:** Intra-and interobserver variability of CMR based measurements for stroke volume.

	Intraobserver Variability		Interobserver variability	
	
	Bias(95% Limits of agreement)*	CoV	Bias(95% Limits of agreement)	CoV
**PA Flow**	-0.8 ± 1.7 (-4.1 to 2.4)	3.1%	-0.9 ± 2.2 (-5.3 to 3.5)	4.0%
**Aorta Flow**	-0.7 ± 2.6 (-5.4 to 5.0)	2.7%	-1.3 ± 2.1 (-4.1 to 5.2)	5.1%
**RV volumes**	-2.1 ± 5.2 (-12.3 to 8.1)	8.7%	-2.6 ± 7.9 (-18 to 13.1)	13.3%
**LV volumes**	-1.8 ± 3.2 (-8.0 to 4.4)	5.4%	-2.6 ± 3.8 (-10.1 tot 4.9)	6.4%

*Bias and limits of agreement were determined according to the Bland and Altman method. CoV = coefficient of variability, PA = Pulmonary artery, RV = right ventricle, LV = Left ventricle.

## Discussion

To our knowledge this study is the first to assess the accuracy of SV derived non-invasively from PA flow, RV volumes, Aorta flow and LV volumes by comparing these values with stroke volume assessed by means of the invasive Fick method in a group of PAH patients.

Our results showed that SV derived non-invasively from PA flow and RV volumes were in poor agreement with the Fick-derived SV. By contrast SV measurement from LV volumes and aortic flow showed good agreement with Fick.

Although SV is highly variable in healthy subjects, an earlier study [18] revealed that SV is fixed in PAH and does not even change during exercise. Thus although the invasive and CMR measurements were not performed synchronously, we expect similar SV values for each patient. The direct Fick principle was chosen as the method of reference because it is considered as a standard for measuring SV in patients with PAH [14].

### SV assessment with PA flow

SV determined from the pulmonary artery flow curve was poorly related to stroke volume assessed by means of Fick. There are several explanations for this discrepancy.

First, we observed non-laminar velocity profiles during systole (figure 1b) in the main PA of PAH patients, which can suggest turbulent or helical flow patterns. Phase-contrast measurements are optimized for laminar flow, whereas in turbulent flow patterns the precision of the CMR flow measurements declines [19,20]. In case of helical flow, motion in the non-velocity encoding directions may also lead to phase-shifts which are not related to through-plane flow.

Second, differences may result from cardiac right-to-left shunting, which causes underestimation of SV determination by PA flow. This is supported by our observation that SV using PA flow showed statistically significant underestimation of 4.2 ml; this underestimation disappeared by excluding those patients with a right-to-left shunt.

Another potential explanation is an inherent inaccuracy of the flow measurement by means of phase-contrast CMR. However, this explanation is unlikely, since in the subset of nine patients where aorta flow was also measured, a much better correlation with the direct Fick is found (figure 6). The accuracy of the aorta flow measurements for SV has already been validated by comparison with invasive SV measurements by Hundley et al [21] in 23 subjects.

### SV assessment with RV volumes

Several factors may explain the errors in the stroke volume measurements derived from the volumetric measures of the right ventricle. First, in the short axis view, in the most basal RV slice, with the RV outflow tract and the inflow region with the tricuspid valve, usually it is more complicated to define the RV contour than in the case of the left ventricle. Because of the large area of this basal slice, errors in choosing the exact RV contour can render inaccurate volume estimates and thus inaccurate SV measurements. Second, evaluation of right ventricular volumes in patients with PAH is difficult because of the complex anatomy of the right ventricle. Third RV endocardial boundary delineation is more complicated because of the considerably more trabeculation of the RV in comparison with the LV [22]. These factors may be improved with further software development to automate boundary detection and also assist in the selection of the basal slice during analyses. Fourth, in patients with pulmonary hypertension tricuspid regurgitation (TR) is common. Thus, with considerable TR, the volumetric SV overestimates the actual SV [23], because it is impossible to differentiate between the volume that moves back through the tricuspid valves and forward though the pulmonary valves.

### SV assessment with LV volumes

The correlation between the SV derived from LV end-diastolic and end-systolic volumes and the SV from Fick was very tight (Figure 5c). This is presumably so because for the LV it is rather easy to delineate the endocardial border and to select the basal slice.

Additionally there was no sign of systematic errors for low or high values indicating that the SV measure from LV volumes is valid for a wide range of SV Values. There were some high SV values in three patients with porto pulmonary syndrome which can be explained by the hyperdynamic circulation feature in these patients [24].

### Intra-observer and inter-observer variability

The results of the current study indicated that the intra- and interobserver variability of SV measurements using PA flow, aortic flow and LV volumes measurements were low, showing small differences and low coefficients of variability. A larger interobserver and intraobserver variability was shown for the RV volumes. This could be explained by factors mentioned above. Together with the poor agreement of RV-derived SV versus Fick, this advises against the use of RV volumes for SV determination.

### Clinical implications

The accurate non-invasive SV assessment has clinical importance because monitoring of SV in PAH patients now becomes feasible as a routine method for monitoring the effects of medical treatment and the follow-up of patients non-invasively. The results of this study have important implications for PAH patients. The SV measurement using PA flow is less accurate in comparison to the SV derived from LV volumes and aorta flow. Therefore, LV volumes and aorta flow should be the methodology of choice in this patients group to measure SV accurately. Consequently, it is advisable to include in the CMR basic protocol a stack of contiguous short axis slices covering the full extend of the left ventricle, and an aortic flow measurement for accurate SV assessment.

## Limitations

We acknowledge some limitations of our study including the fact that catheter studies and CMR examinations could not be performed simultaneously. However, the results of this study demonstrate that SV derived from LV volumes correlate well with measurements obtained using the direct Fick method, supporting earlier findings that stroke volume does not vary over a short period of time in PAH, making this parameter of value to monitor PAH patients.

Furthermore we acknowledge the small sample size of the aorta flow measurements.

Finally, the present study does not provide conclusive evidence of the cause of the discrepancy between the PA flow and the direct Fick.

## Conclusion

In patients with PAH, taking the direct Fick as a standard invasive method, non-invasive derivation of SV should preferably be carried out using aortic flow or LV volumes. SV assessment with PA flow or RV volumes shows limited accuracy.

## Abbreviations

CO: cardiac output; CoV: coefficient of variability; EDV: end diastolic volume; ESV: end Systolic volume; LV: left ventricular; CMR: cardiovascular magnetic resonance imaging; PA: pulmonary artery; PAH: pulmonary arterial hypertension; RHC: right heart catheterisation; RV: right ventricular; SV: stroke volume.

## Competing interests

The authors declare that they have no competing interests.

## Authors' contributions

GM participated in data collection, study design, data analysis and interpretation and manuscript preparation. JTM participated in data collection, study design, interpretation and manuscript revision. AB participated in interpretation and manuscript revision. PEP participated in manuscript revision. NW participated in interpretation and manuscript revision. AVN participated in study design, interpretation and manuscript revision. All authors read and approved the manuscript.
